# Peptides, Antibodies, Peptide Antibodies and More

**DOI:** 10.3390/ijms20246289

**Published:** 2019-12-13

**Authors:** Nicole Trier, Paul Hansen, Gunnar Houen

**Affiliations:** 1 Department of Neurology, Rigshospitalet Glostrup, Nordre Ringvej 57, 2600 Glostrup, Denmark; nicole.hartwig.trier@regionh.dk; 2 Department of Drug Design and Pharmacology, Universitetsparken 2, 2100 Copenhagen Ø, Denmark; prh@sund.ku.dk

**Keywords:** antibodies, aptamers, peptides, peptide antibodies, recognition molecules, synthetic libraries

## Abstract

The applications of peptides and antibodies to multiple targets have emerged as powerful tools in research, diagnostics, vaccine development, and therapeutics. Antibodies are unique since they, in theory, can be directed to any desired target, which illustrates their versatile nature and broad spectrum of use as illustrated by numerous applications of peptide antibodies. In recent years, due to the inherent limitations such as size and physical properties of antibodies, it has been attempted to generate new molecular compounds with equally high specificity and affinity, albeit with relatively low success. Based on this, peptides, antibodies, and peptide antibodies have established their importance and remain crucial reagents in molecular biology.

## 1. Introduction

Peptides and antibodies (Abs) have entered a fruitful companionship in immunology since they were discovered. Peptide chemistry formed the basis of understanding protein composition and structure and Abs lay the foundation for molecular immunology, even though the relationship between Abs and antigens (Ags) had to await advances in peptide and protein chemistry. These advances led to the realization that Abs and a major group of Ags are themselves proteins [1,2]. Peptides were also crucial reagents for elucidating the molecular biology of Ab specificity and biosynthesis, both with regard to B cell specificity and development and with regard to antigen presentation and T cell specificity and development [1,2].

Today, molecular biology still depends on the use of peptides, Abs, and peptide Abs. This applies to research and diagnostics but also to therapy and may become relevant to prevention of disease (vaccination). In addition, new molecule types are being developed to complement the use of the traditional reagents and these may become more useful if the technologies can be improved.

## 2. Peptides

### 2.1. Peptide Discovery

**Figure 1:**
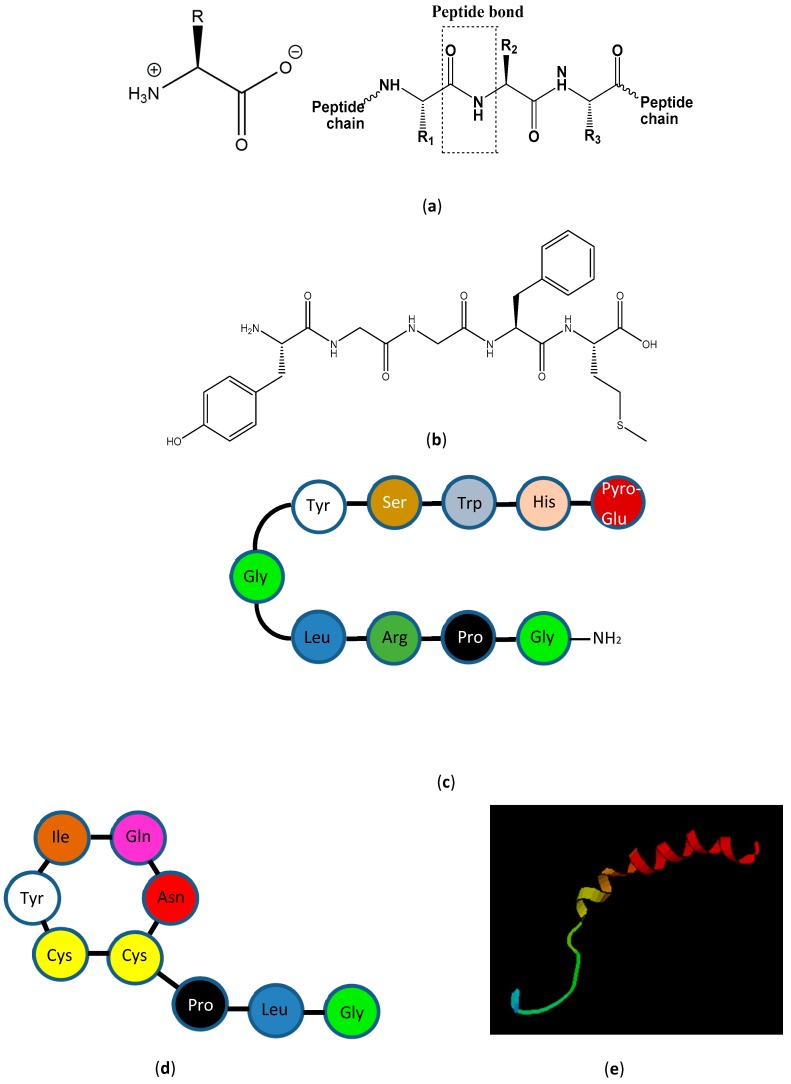
Amino acids, peptide bonds, polypeptides, and proteins. (**a**) Amino acid and peptide bond structure. The box indicates a peptide bond (-CO-NH-). (**b**–**e**) Examples of smaller bioactive peptide hormones also illustrating particular conformational aspects. (**b**) Met-enkephalin, a non-structured opioid penta-peptide. (**c**) Luteinizing hormone releasing hormone, a β-strand deca-peptide hormone. (**d**) Oxytocin, a small disulfide bridge-constrained uterus-contracting nona-peptide hormone. (**e**) Neuropeptide Y, a 36-amino acid peptide hormone containing an α-helix. Figure 1e is obtained from https://commons.wikimedia.org/wiki/File:Neuropeptide_Y.png.

The history of peptide chemistry dates back to around 1900, where Emil Fischer synthesized small peptides containing glycine residues [3]. The field slowly developed by introducing protecting groups for the Nα-amino group [4] and side-chain functional groups [5] as well as more effective coupling reagents for peptide bond formation [6]. In 1953, Du Vigneaud and co-workers synthesized the first biologically active peptide, oxytocin, a uterus-contracting hormone containing nine amino acids and a disulfide bond [7], as shown in Figure 1 together with other examples of bioactive peptides. Further advances in the field included Edman degradation and amino acid analysis with the former being a method for sequencing a peptide one N-terminal residue at a time [8]. Protein sequencers with Edman degradation became available in the late 1960s [9], and ninhydrin-based amino acid analysis was introduced by Moore and Stein who elucidated the structure of ribonuclease A in 1973 [10].

In 1963, Robert Bruce Merrifield introduced the solid-phase peptide synthesis (SPPS) principle, in which a growing peptide chain is linked through the C-terminal end to a solid-support [11]. Previously, peptides were synthesized in solution and purified after each coupling step. In SPPS, the peptide chain is elongated toward the N-terminus in a step-wise manner using a protecting group for the Nα-amino group and semi-permanent groups for side chains [11]. Following synthesis, the peptide is cleaved from the solid-support with acid.

From here on, the maturation of the field was mainly driven by the introduction of analytical and preparative reversed-phase high-performance liquid chromatography [12] and mass spectrometry (MS) techniques such as matrix-assisted linear desorption-ionisation Time-Of-Flight, MALDI TOF MS [13], and liquid chromatography, LC-MS [14], which made it possible for most laboratories to purify and characterize their peptide products.

### 2.2. Peptide Synthesis

The most widely used method for chemical synthesis of peptides is 9-fluorenylmethyloxycarbonyl (Fmoc) SPPS [15]. In this method, the Nα protecting group is Fmoc and acid-labile tert-butyl-based groups are used for side chain protection. Formation of the peptide bond is facilitated by an auxiliary nucleophile such as 1-Hydroxy-7-azabenzotriazole, HOAt, and an in situ coupling reagent such as O-(7-Azabenzotriazol-1-yl)-N,N,N′,N′-tetramethyluronium hexafluorophosphate, HATU. This technology has been refined, so that today it is possible to synthesize almost any peptide of interest [16]. Larger proteins, up to 350 amino acids, may be synthesized by native chemical ligation, introduced by Kent and coworkers in 1994 [17] and reviewed recently [18]. Native Chemical ligation is also useful for introducing non-proteinogenic amino acids and labelling of proteins. However, proteins are most efficiently made by recombinant technology.

### 2.3. Properties

The biological activity of a peptide is coupled to its conformation, i.e., the essential functional groups must be in a required spatial orientation [19]. Peptides can adopt different secondary structures such as α-helix, β-sheet, hairpin, and random coil (Table 1), which are stabilized by hydrogen bonding, electrostatic and hydrophobic interactions, disulfide bonds, and/or cyclization.

α-helical or β-structures may form amphipathic structures in which the polar side chains are segregated on one face of the structure and hydrophobic side chains are on the other [24]. Amphipathic structures are important to the biological activity of smaller peptides, which was first realized by Kaiser [25]. The structure of peptides is typically studied by circular dichroism spectroscopy [26], nuclear magnetic resonance spectroscopy [27], electron microscopy [28], or X-ray crystallography [29].

### 2.4. Applications

Synthetic peptides have numerous applications in research, diagnostics, and treatment [30,31], epitope characterization [31,32,33,34], production of peptide Abs [35], and vaccine development [36] (Table 1). Synthetic peptides are important therapeutics, and, in the last two decades, a significant renaissance in peptide drug discovery has occurred [37,38]. Since 2000, 28 new peptide drugs have been approved for a wide range of conditions, cancer, infections, metabolic diseases, haematology, cardiovascular diseases, and osteoporosis [39].

One example of the use of synthetic peptides in diagnostics is the detection of Dengue virus Abs, as described by Bergamaschi et al. in this special issue [40] (Table 2). They successfully used a peptide representing a predicted immune-dominant (linear) epitope in Dengue Virus Envelope protein to detect Abs in infected individuals. Another particularly illustrative example of the use of peptides in diagnostics is the highly sensitive and specific detection of anti-citrullinated protein Abs, associated with rheumatoid arthritis, using synthetic peptides containing citrulline, which in vivo arises by post-translational modification (PTM) of Arg [31]. This also illustrates that peptides continue to be important reference compounds, especially with regard to PTMs. This requires further developments of synthetic strategies.

## 3. Antibodies

### 3.1. Discovery

**Figure 2:**
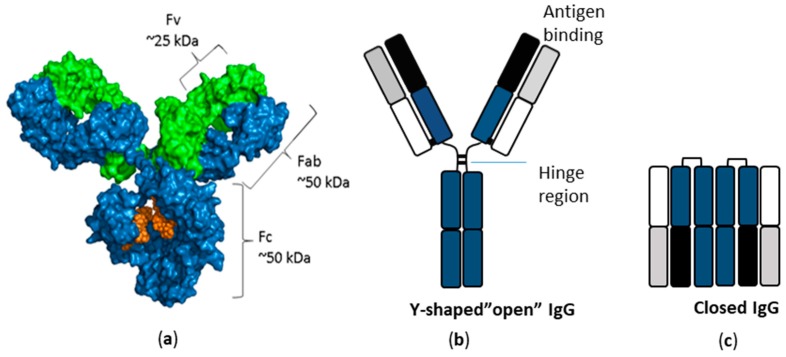
Schematic illustration of antibody structures. (**a**) Space filling model of an IgG. (**b**) IgG structure presented as a classical Y-shaped structure. (**c**) IgG structure presented as a compact (“closed”) structure.

Abs, which are also referred to as immunoglobulins (Igs), are an indispensable part of the human immune system and occur in different forms, denoted IgM, IgD, IgG, IgA, and IgE (Figure 2) [1,2]. They were discovered during the late 19th and early 20th century as substances that react with Ags, but it was only later that it was realised that they are proteins [1,2,55]. More specifically, Abs are large symmetric heterotetrameric glycoproteins composed of two identical heavy chains and two identical light chains (Figure 2).

The covalent structure of Abs was gradually elucidated by Edman degradation during the first part of the 20th century [56] and the three-dimensional structure of Abs was characterised by crystallography in the second part of the century [57,58,59]. Traditionally, the structure of Abs has been illustrated by models as Y-shaped molecules (as shown in Figure 2). X-ray structures of Abs have mainly been limited to isolated Fab and Fc parts, even though a few structures of whole Abs have been published [60,61]. However, recent results using ion exchange-purified IgGs and chemical cross-linking in combination with MS have shown that native IgGs have a compact (“closed”) structure [62].

Abs interact with Ags through their antigen-binding sites located at the tip of the Fab “arms.” This interaction is mediated by a multitude of co-operative specific interactions, which makes antibodies highly conformation-specific. Due to the structure of the antigen-binding sites and the ability of Ab-producing cells (B cells) to undergo affinity maturation, the potential for antigen interaction is essentially unlimited [1,2]. For this reason, antibodies have become essential tools in molecular biology, diagnostics, and therapy (Table 3), which is a development catalysed by advances in Ab production and characterisation technologies.

### 3.2. Polyclonal Antibodies

Polyclonal antibodies (PAbs) are derived from human or animal sera, which may be used in crude form or after purification (ammonium sulfate precipitation, ion exchange chromatography, protein A/G affinity chromatography, ligand affinity chromatography, etc.) [85,86]. As such, PAbs either represent naturally occurring Abs or Abs induced upon immunization. Basically, PAbs are produced by injecting an immunogen into an animal along with an adjuvant [87]. Adjuvants are used in the immunization to enhance the immune response [88,89]. Several studies have been conducted to identify the most efficient adjuvant. Aluminumhydroxide, Al(OH)_3_, is often favoured since it has relatively few side effects and effectively activates the innate immune system, especially the alternative complement pathway, which leads to opsonisation and activation of the adaptive immune system and, ultimately, to production of Abs with high titres [88,89]. The most common host animal for production of PAbs is the New Zealand white rabbit, but larger animals (e.g., goat, sheep, and horse) are often used for large scale production [87]. Subcutaneous injections are preferred, since they are effective and least harmful to the animal. After being injected with a specific immunogen, the animal is usually given further immunizations (boosting) to ensure Abs of high titers against the specific target [87]. After immunization and boosting, PAbs are obtained, while recognizing different epitopes of an antigen. Upon prolonged immunization, specific clones may become dominant, which increases the specificity of the Abs, and immuno-affinity purification may be used to obtain monospecific PAbs [90]. In the case of a natural infection, the spectrum of PAbs can be characterised by proteomic methods, e.g., after isolation of IgG, as illustrated by Lundström et al. in this special issue (SpotLight proteomics) [91] and used for production of recombinant Abs. Table 3 illustrates therapeutic PAb products obtained from human sera or by immunization of animals. Prominent examples are Rhesus-D PAbs (RhD Ig) used for rhesus syndrome prophylaxis and anti-botulinum toxin PAbs used for treating intoxications [69,70,71].

### 3.3. Monoclonal Antibodies

PAbs may exhibit unwanted reactions, including cross-reactivity, and, for many purposes, more well-defined Abs are desirable. Furthermore, PAbs may be challenging to reproduce, as they have a higher batch-to-batch variation and, therefore, require thorough characterization before use. Köhler and Milstein pioneered the technique for producing monoclonal Abs (MAbs) of desired specificities [92,93]. The initial steps for generation of PAbs and MAbs are similar [86,87,94]. The immunogen is injected into the host along with an adjuvant, which is followed by repetitive immunizations to boost the immune response of the immunized animal. As described above, the Abs generated are polyclonal, and, if aiming for MAbs, isolated B cells from the immunized animal are fused with myeloma cells. Single clones producing a sufficient amount of Abs are identified by screening and expansion [94]. The main advantage of isolating clones is that MAbs may be produced in unlimited quantities. This technique is still the major method for MAb production, as described by Köhler and Milstein, even though minor modifications may be introduced, mainly in the screening phase, where an assay system identical to the end stage use should be used to avoid selection of clones with aberrant properties [94]. Upon successful screening and cloning, highly specific MAbs are obtained and the epitopes of these can be characterised with high precision, as illustrated by recent studies of MAbs to the glutamate decarboxylase isoforms 65 and 67, which were shown to recognise linear epitopes located in flexible structures [47,95]. Similar results have been obtained with many other MAbs, which verifies the stringent requirements for an optimal antibody-antigen interaction [33,46,96,97]. MAbs may be used as crude culture supernatants (which usually contain bovine Ig) or after purification, e.g., on protein A/G [94,98] or synthetic resins, as illustrated by Islam et al. in this issue [90]. However, it is important to remember, that such purification methods may not remove the bovine IgG, which originates from the cell culture medium [99,100]. This is an issue that is mostly ignored or over-looked. Murine MAbs have had a few therapeutic uses (e.g., Muromonab anti-CD3 [101]), but due to high immunogenicity have lately been replaced by humanised or fully human recombinant MAbs.

MAbs may be produced in forms of humanized or chimeric antibodies. A chimeric antibody is an antibody made by fusing the antigen binding region from one species with the constant domain from another species [102,103], whereas humanized antibodies are generated by replacing the hypervariable loops of a fully human antibody with the hypervariable loops of a murine antibody [103,104]. Chimeric antibodies are 70% human and contain a complete human Fc portion, which makes them considerably less immunogenic in humans. Humanized antibodies are 85% to 90% human and are even less immunogenic than chimeric antibodies. However, generation of humanized antibodies is technically demanding when compared to the generation of murine antibodies. Most of the approved mAbs for therapeutic use are either chimeric or humanized (Table 3).

### 3.4. Recombinant Antibodies

Advances in molecular biology techniques, including sequencing, oligonucleotide synthesis, proteomics (incl. antibody proteomics), etc., have made it possible to produce recombinant Abs with pre-determined properties [105,106,107]. However, even today, most recombinant Abs represent variants of traditional MAbs, since ab initio isolation of Abs from “naïve” libraries usually yields MAbs with rather low affinities. Instead, designed libraries are usually produced and screened for Abs with desired properties. Examples of recombinant Abs are numerous, including therapeutic Abs and various immunoassays [108,109,110,111]. Table 3 shows examples of therapeutic Abs for treating human diseases. Therapeutic Abs have had enormous success, exemplified by Rituximab (an anti-CD20 MAb), which is the first therapeutic MAb for treating lymphoma and used for treatment of several autoimmune diseases [112]. Other notable examples are recombinant MAbs to tumour necrosis factor, TNF, and programmed death-1, PD-1, used for treating rheumatoid arthritis and malignant melanoma, respectively [74,75]. Recombinant Abs may be produced in various systems, including mammalian cell lines, yeasts, and phage display [90,113]. For phage display, engineered single chain fragment variable (scFv) antibodies are a preferred format due to the reduced size, which makes expression and production easier. Affinity maturation of such scFvs is feasible by various methods, as described by Lim et al. in this issue, but may require substantial work before Abs with the desired properties are obtained [114].

### 3.5. Engineered and Designed Antibodies

The advances in recombinant antibody technology described above have also made it possible to engineer Abs for specific purpose, including Abs with increased stability, Abs without effector functions or with reduced/increased effector functions (Fabs, scFvs, Ab-drug conjugates, etc.), Abs with several specificities (bispecific, tri-specific, etc.) [110,115,116,117]. Abs have also been used to convey particular specificities to effector T cells with good effects in the form of chimeric antigen receptor T cells (CARTs) [118]. Alternative “antibody-like” binders based on protein scaffolds have been developed in the form of designed ankyrin repeat proteins (DARPins), anticalins, affibodies, knottins, and others [119,120,121,122,123]. These binders rely on the same principles as antibodies for target recognition and may be further developed, and become more important in the future, since their affinities and specificities may approach those of antibodies and since they may have properties complementing those of antibodies.

A separate class of (designed) Abs with predetermined specificity are peptide Abs, which may be either polyclonal or monoclonal.

## 4. Peptide Antibodies

### 4.1. Discovery and Properties

Peptide Abs were originally described in the 1980s, where peptides coupled to a carrier protein were used for generating peptide-specific Abs [124]. This approach was used in the following decades, primarily to increase the general understanding of antigenicity and immunogenicity [125,126].

What basically separates traditional Abs from peptide Abs is the immunogen used for immunization. Thus, peptide Abs are strong competitors to traditional Abs, since these Abs, in theory, can be directed to any peptide, even with small molecular differences [127]. Typical peptide antibodies are directed to PTMs, conserved regions, intra-cellular or extra-cellular domains, cleavage sites, tags, and specific conformations, which can be rather challenging to control when using proteins for immunization [128]. Furthermore, peptide Abs may recognize native as well as denatured proteins with high specificity, and may even be directed to toxic or hazardous proteins, which are difficult to purify and to use in traditional antibody production [129]. Peptide Abs are usually of high specificity and affinity, and have the advantage that the antigenic target is already well-defined [94,128,129]. Based on this knowledge, peptide Abs have become a powerful tool, not only for immunological research approaches but in clinical diagnostics as well [66,67,128,130,131].

### 4.2. Production

Traditional peptide antibody production is based on immunization, where a crucial element is the peptide selection. Peptides used for immunization are usually 10–20 amino acids long, and peptides below 10 amino acids and above 20 amino acids are usually not preferred, since these peptides may elicit Abs that do not recognize the protein with sufficient affinity or specificity [46,130,132,133,134]. Algorithms are available for peptide selection even though these are based on peptide antigenicity rather than immunogenicity [128]. Peptides originating from protruding regions, exposed termini and flexible regions such as turns, loops, and connecting regions that are often favoured in the same manner, and regions with Pro and Gly may be favoured, since they are often represented in these structures [33,128,129,135,136,137]. A Cys residue in the N- or C-terminus of the peptide is optimal for conjugation of the peptide to a carrier [134]. However, two Cys residues or more should be avoided due to the potential of disulfide bond formation, unless deliberately used for specific conformational reasons (Table 4).

Following peptide synthesis, the peptides are conjugated to carriers to ensure immune responses with high antibody titres, since many peptides are not immunogenic by themselves. Traditional carriers include bovine serum albumin, keyhole limpet hemocyanin, and ovalbumin [45,128,139,140,141]. The selected peptide and the carrier are conjugated using bifunctional reagents such as glutaraldehyde, carbodiimide, N-succinimidyl 3-(2-pyridyldithio)propionate, or 3-maleimidobenzoic acid N-hydroxysuccinimide ester, which have been described elsewhere [35]. Upon immunization, the peptide-carrier conjugates are mixed with the adjuvant and injected into the selected animal, which is most often a mouse or rabbit. Following repetitive boosts, it may be necessary to screen for the selected target [87,94]. Although peptides of 15–20 amino acids are often applied, an epitope is usually 5–8 amino acids long. Therefore, a peptide of 15–20 amino acids may, in theory, give rise to an antibody response recognizing more than one epitope [87,94]. As a consequence, it may be necessary to characterize the different MAbs to ensure a specific reactivity, e.g., to a central modified amino acid. Otherwise, the Abs obtained may be used in the crude form [87,94]. This may be circumvented by using shorter peptides, even though this may result in lower Ab titers and reduced immunogenicity. If the peptide contains a terminal Cys residue, the peptide is easily immobilized and used in affinity chromatography to purify the Abs. To avoid selecting for peptide Abs that do not recognize the final target in the intended assay, antibody screening should be conducted in the assay that the Abs are to be used in [94,128,129,137,142].

### 4.3. Applications

Currently, peptide Abs are used in a variety of immunoassays such as immunoprecipitation (IP), sandwich assays, immunoblotting, immunocytochemistry, and immunohistochemistry (IHC). Applications range from powerful research tools to specific diagnostic markers [128].

Peptide Abs are used in the diagnostic field in a variety of assays to aid the diagnosis of infection and diseases, for precise quantification or to identify or locate the presence of a given substance [128]. In enzyme-linked immunosorbent assays (ELISA)s, peptide Abs are usually used as detecting antibody in indirect ELISA or as a capture antibody in sandwich ELISA. Furthermore, peptide Abs are often used for microscopic examination of tissues by IHC, where binding of the antibody to the final target is visualized by enzymatic methods or by fluorescence.

Peptide Abs to cell surface markers are often used at fluorescent conjugates in fluorescence microscopy for IHC or flow cytometry (FC) for cells in suspension [63,64,65,143].

Detection of native targets using peptide Abs is primarily conducted using FC, where Abs are used to count and separate cells, and by IP. Furthermore, the use of peptide Abs to denatured targets often applies to localization, quantification, and detection of targets in IHC, ELISA, and Western blotting [128].

Peptide Abs have been employed in IHC for detecting specific mutations with great success. These mutation-specific peptide Abs are used in the diagnostics of various cancers to determine the presence of single point mutations or deletions in B-Raf or isocitrate dehydrogenase, associated with metastatic melanoma and the epidermal growth factor receptor, which is associated with melanoma, glioma, and lung adenocarcinoma, respectively [44,66,68]. Besides being used in the detection of various cancer types, peptide Abs have, among others, been used for diagnosing neurodegenerative diseases, infectious diseases, disorders of the immune system, cardiovascular diseases, and other conditions [128]. Moreover, peptide Abs and peptides are used to detect viruses, bacteria, and parasites and Abs to these [128].

Peptide Abs are used for numerous research purposes as well. For example, peptide Abs are often used for detecting cell surface markers, e.g., CD3, CD8, CD14, CD20, and CD34 in FC [63,64,65,143].

Despite obvious potential, peptide vaccines or peptide Abs have not yet made it into clinical therapeutic use. One of the first descriptions of peptide Abs was on hand foot and mouth (HFM) virus [124] and efforts are still ongoing to develop peptide-based vaccines targeting the many different enteroviruses capable of causing HFM disease, as described by Anasir and Poh in this issue [144].

As mentioned earlier, therapeutic MAbs have revolutionized treatment of many serious diseases. This would be expected to lead to a breakthrough for peptide vaccines, i.e., by inducing Abs to known therapeutic targets by vaccination (e.g., CD20, TNF, and PD1). However, this expectation has not been fulfilled yet. Rituximab targets an epitope on the extracellular domain of CD20, which is a prominent B cell antigen, and is an attractive target for a peptide vaccine inducing CD20 Abs. However, despite several attempts, no such vaccine has been successful as described by Favoino et al. in this issue [145]. One of the problems with this approach is that it is difficult with a synthetic peptide to mimic the three-dimensional structure of native membrane-bound CD20. Thus, even though the synthetic peptides induce Abs, they are not necessarily reactive with the target antigen, which testifies to the very high specificity of Abs. The same applies to putative mimotopes of CD20, which may be identified by phage display screening for CD20 epitopes/mimotopes, but do not elicit CD20 cross-reactive Abs upon immunization [145], which, again, demonstrates the very high specificity of (peptide) Abs.

Another potentially promising use of peptide vaccines or therapeutic peptide Abs is Alzheimer’s disease, which is believed to be caused by accumulation of toxic amyloid β aggregates in the brain [146,147]. This aggregate is composed mainly of residues 1-42 of the amyloid precursor protein, and is released by proteolytic cleavage, where it accumulates in brain plaques [146,147]. Due to the small size of amyloid β, it is an attractive candidate for a peptide vaccine or a therapeutic MAb, but efforts along this line have not led to new treatments yet.

Similar to amyloid β, α-synuclein is believed to be pathogenic in Parkinson’s disease, where it accumulates intracellularly in brain neurons [148]. In analogy with Alzheimer’s disease, immunotherapy has been proposed as a possible therapy for Parkinson’s disease. In this issue, Shen et al. describe production of MAbs to various fragments of α-synuclein and show that residues 15-65 not only self-aggregate but also promote aggregation of full length α-synuclein. Furthermore, a MAb targeting this fragment diminished aggregation of α-synuclein [149].

## 5. Alternative Recognition Molecules for Antigen Targeting

Despite their versatility and many excellent properties, Abs also have some inherent limitations. Due to their considerable size and specific properties, unwanted side reactions may cause problems in some applications, e.g., ELISA (non-specific binding) and IHC (low tissue penetration, non-specific binding). This can, to some extent, be relieved by the use of blocking reagents and by the use of recombinant (single chain) antibody fragments. Abs also have a potential for cross-reactions, which is a phenomenon that is not well described/understood except for a general theory of molecular similarity/mimicry [150,151,152,153,154].

Due to the limitations/problems of Ab production and applications, considerable efforts have been done to develop alternative recognition molecules and libraries. The types of recognition molecules and libraries that have been investigated include peptide libraries and libraries of AA-like monomers, dendrimers, aptamers, molecular imprints, and others (Table 5).

### 5.1. Peptide Libraries

Several types of peptide libraries have been developed for various screening purposes [155,156]. Combinatorial peptide libraries have been used for basic research and drug discovery, although with mixed success. Positional scanning libraries have large diversity, but often do not yield specific recognition peptides, presumably due to a large flexibility of the peptides. However, such libraries have been used to identify a range of protease substrates and protease inhibitors as well as optimal B and T cell epitopes [167,168,169,170,171]. One advantage of such libraries is that D-amino acids, non-natural amino acids, or other types of molecules, e.g., PTMs, can fairly easily be incorporated, which sometimes yields peptides with improved properties [167,172].

### 5.2. Dendrimers

Dendrimers are branched molecules with a predefined structure usually based on repetitive addition of monomer building blocks to a central core molecule and can be thought of as three-dimensional analogues of linear molecules. They should, in principle, be capable of yielding specific recognition molecules, depending on the diversity of the monomer building blocks [160,161]. In practise, however, dendrimers have found relatively few applications, possibly due to low affinity and steric hindrance effects.

### 5.3. Aptamers

**Figure 3:**
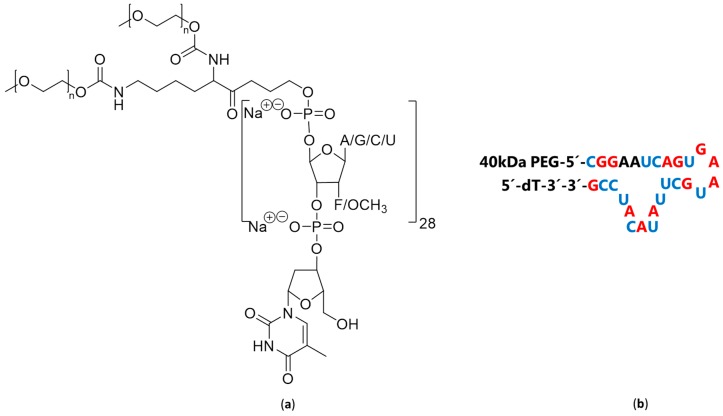
Chemical structure and Predicted secondary structure of Pegaptanib. (**a**) Chemical Structure of Pegaptanib. (**b**) Predicted secondary structure of Pegaptanib. Modifications 2′-fluoropyrimidines shown in blue and 2′-methoxy-purines shown in red.

Aptamers are single chain nucleotides (RNA, DNA, and XNA (xeno-nucleic acids)) with a well-defined structure due to intramolecular base pairing, and such molecules can be amplified and selected from various libraries using polymerase chain reaction (PCR) technologies and affinity selection [173,174,175,176,177]. A much-used method is SELEX (systematic evolution of ligands by exponential enrichment), which amplifies aptamers from random libraries by repetitive rounds of affinity selection (panning) combined with PCR amplification. However, aptamers amplified by this method usually requires further optimization in order to become practically useful, which is also testified by the relatively low number of currently useful aptamers [170,178]. One aptamer, which has found clinical use, is Pegaptanib, which is a vascular endothelial growth factor (VEGF)-targeting aptamer (Figure 3) used for treatment of various forms of pathological ocular neovascularization [166]. The success of this aptamer may relate to an ability to interact with the heparin-binding site of VEGF by mimicking the repetitive negative charges on heparin.

### 5.4. Other Recognition Molecules

Molecular imprinting is a technique where polymers are assembled on molecular templates, which are subsequently removed, which leave a “molecularly imprinted” polymer. This can, in principle, be used as antibody mimic, receptor mimic, or, in general, a molecular sensor [164,165]. Imprinted polymers can be made on the basis of a wide range of (polymerizable) monomers and can be made and further modified “post-imprinting” to have Ab-like recognition properties [162,163]. Compared to Abs, they are relatively cheap and easy to produce, but cannot yet compete with antibodies in terms of affinity and specificity. Practical applications are therefore essentially missing at present.

## 6. Discussion and Conclusions

Peptides are widespread in nature and have numerous biomedical applications for diagnostics, therapy, and research. With 20 amino acid building blocks and many PTMs, the diversity of peptides is extremely large, which is also testified by the number of different bioactive naturally occurring peptides. Peptides are therefore extremely important in all fields of molecular biology and medicine, not only in the form of natural or synthetic agonists and antagonists, where specificity is determined by conformational or disulfide bridge-dependent sterical constraints, but also in clinically applicable platforms based on f. ex. short cysteine-constrained recognition scaffolds, where the specific antigen or receptor recognition is enabled by the constraint. One of the most important uses of peptides is for the production of peptide Abs. Peptides have very low immunogenicity themselves but can be used for induction of Abs, when coupled to a carrier protein, which can provide T cell epitopes for induction of Abs. The conjugation also limits the conformational flexibility of the peptides, which makes them more immunogenic/antigenic. Due to their structure and composite antigen binding sites (complementarity-determining regions), Abs are very specific recognition molecules and interact with their epitopes through multiple bonds, which gives them high affinity and specificity. Moreover, they can be obtained fairly easily and go through multiple rounds of affinity maturation during immunization, which makes them highly valuable and, until now, irreplaceable reagents for diagnostics and therapy. The advantage of using peptide antibodies are that they, in theory, can be directed to any target. However, peptide antibodies may be difficult to generate with sufficiently high titers. Moreover, they do not necessarily recognize the native antigen. Similar when generating MAbs to a protein, it may be difficult to generate an antibody to a specific site, e.g., a modification or a specific domain. These obstacles when using MAbs and peptide antibodies, may be avoided by screening for reactivity to targets in the intended assay.

A notable limitation when using Abs is tissue penetration both in vitro (e.g. IHC) and in vivo. For example, MAbs directed to e.g., tumor-specific antigens often remain in the blood and only approximately 20% of the administered dose typically interacts with the tumor [179]. Moreover, MAbs can have various modes of action, and the actual mode of action once administered to patients is not always clear [119]. Another limitation when producing antibodies for therapeutic uses are the production costs. The use of a very large culture of mammalian cells and extensive purification steps limit the use of antibodies. Another naturally inherent limitation of natural and synthetic Abs for therapy is the ability of pathogens to mutate and evade antibody responses and the inherent immunogenicity of proteins, even in the form of fully human or humanized Abs. Abs also have some inherent limitations due to size and other physical properties, and, therefore, many attempts have been made at developing other molecule types with equally high affinity and specificity. Alternative protein-based scaffolds relying on Ab-derived principles for selection have been developed and are beginning to complement Abs. However, in terms of therapy, such binders can be expected to suffer from equal or even higher problems with immunogenicity as Abs. The most successful non-protein recognition molecules developed to date are the aptamers, self-folding RNA molecules, which can be selected and amplified from various libraries, but usually require further modifications in order to become useful. However, despite much research and development, very few aptamers are of commercial importance today.

In conclusion, peptides, Abs, and peptide Abs currently remain the cornerstone reagents in molecular biology, which is also illustrated by several papers in this issue. Hopefully, in the future, new and more advanced recognition molecules will be generated, which may complement Abs and have diagnostic as well as therapeutic uses.

## Figures and Tables

**Table 1:** Representative peptides with a secondary structure and their biological activity.

Name	Sequence	Biological Activity	Secondary Structure	References
Calcitonin- Gene Related Peptide	ACDTATCVTHRLAGLLSRSGGVVKNNFVPTNVGSKAF	Vasodilator, Migrane	α-helix	[20]
Gramicidin S	cyclo[D-Phe-Pro-Val-Orn-Leu]_2_	Antimicrobial	Antiparallel β-sheet, cyclic	[21]
Peptide 2	KHQCHWECT-Cit-GRCRLVCGRSGS	Reacts with rheumatoid autoantibodies	β-hairpin, disulfide bonds (C^4^–C^17^, C^8^–C^13^)	[22]
DX600	GDYSHCSPLRYYPWWKCTYPDPEGGG	Inhibitor of angiotensin converting enzyme 2	Random coil, disulfide bond (C^6^–C^17^)	[23]

Unusual amino acids: Orn: Ornithine. Cit: Citrulline.

**Table 2:** Examples of peptide applications in different areas of molecular science.

Area	Examples	References
**Research**		
Protease substrates	γ-secretase TM peptide substrates/AD	[41]
Protease inhibitors	HIV-protease inhibitors/HIV subtype C	[42]
Cell adhesion	N-methylated TSP-1 peptides/CLL	[43]
Peptide antibody production	P110/ mycoplasma genitalium, b-raf/ malignant melanoma	[44,45]
Epitope identification	GAD/ diabetes, CENPF/ cancer, NMDAR/ encephalitis	[34,46,47]
**Diagnostics**		
Antibody detection	ACPA/RA, Gliadin/CD, DVEP/Dengue fever	[31,40,48,49,50]
Peptide quantification	Insulin/diabetes, C peptide/diabetes	[51]
**Therapeutics**		
Peptide drugs	Leuprolide/cancer, desmopressin/diabetes	[52,53]
Vaccine development	Cell-penetrating peptides/DNA vaccine delivery	[54]

ACPA: anti-citrullinated protein antibody. AD: Alzheimer disease. CD: celiac disease. CENPF: centromere protein F. CLL: chronic lymphocytic leukemia. DVEP: Dengue virus envelope protein. GAD: Glutamate decarboxylase. NMDAR: N-methyl-D-aspartate receptor. HIV: human immunodeficiency virus. RA: rheumatoid arthritis. TM: transmembrane. TSP-1: thrombospondin-1.

**Table 3:** Examples of antibody applications in different areas of molecular science.

Antibodies	Examples/Uses	Antibody Type	References
**Research**	**Target Recognition**		
	CD14, CD20, CD34/ELISA, ICC, WB, FC	Peptide Ab (MAb)	[63,64,65]
**Diagnostics**	**Target Quantification**		
	Isocitrate dehydrogenase, B-Raf, Epidermal growth factor receptor/IHC	Peptide Ab (MAb)	[66,67,68]
	melanoma Glycoprotein B, Herpes simplex encephalitis/IHC, ELISA	Peptide Ab (MAb)	
**Therapeutics**	**Target Neutralization**		
	Rhesus-D Ig/rhesus syndrome prophylaxis	PAb (Sp IVIG)	[69]
	Anti-toxins/botulism	PAb (Sp IVIG)	[70,71]
	Rituximab/lymphoma, Ocrelizumab/multiple sclerosis	MAb (recombinant), chimeric	[72,73]
	Infliximab/rheumatoid arthritis	MAb (recombinant), chimeric	[74]
	Nivolumab/malignant melanoma	MAb (recombinant), human	[75]
	Panitumumab/EGFR metastatic colorectal carcinoma	MAb (recombinant), human	[76]
	Daclizumab/allograft rejection	Mab (recombinant), humanized	[77]
	HpHbR Ab-PBD conjugate/African trypanosomiasis treatment (mouse model)	MAb-drug conjugate	[78]
**Vaccines**			
	MMR live attenuated viruses vaccine/measles, mumps, rubella prophylaxis	PAb in vivo	[79]
	DiTePePolHiB SU vaccine/diphtheria-tetanus-pertussis-polio-hemophilus prophylaxis	PAb in vivo	[80,81,82]
	HPV SU vaccine/cervix cancer prophylaxis	PAb in vivo	[83,84]

CD: cluster of differentiation. DiTePePolHiB SU: Diphteria-Tetanus-Pertussis-Polio-Hemophilus influenzae B subunit vaccine. EGFR, epidermal growth factor receptor. ELISA: enzyme-linked immunosorbent assay. FC: flow cytometry. HpHbR: haptoglobin-hemoglobin receptor. IVIG: Intravenous immunoglobulin. HPV: human papilloma virus. ICC: immunohistochemistry. IHC: immunohistochemistry. MAb: monoclonal antibody. MMR: Measles-mumps-rubella. PAb: polyclonal antibody. PBD: pyrrolobenzodiazepine. Sp: Specific. SU: subunit. WB: Western blotting.

**Table 4:** Peptide antibody selection and applications.

Selecting Factors	Examples	References
Amino acid composition	Hydrophilic aas, charged aas, Pro, Gly (represented in loops)	[33,138]
Peptide length	8–25 aa	[46,130,132,133,134]
Peptide structure	Linear, flexible, cyclic, loops, turns, helices	[25,86,87,93,94,95]
Protein target	Accessible epitope, areas of high conservation, areas of hypervariability, N/C-termini, post-translational modifications	[128,129,138]

**Table 5:** Examples of synthetic recognition molecules for antigen targeting.

Area/Molecule Types	Examples/Uses	References
**Research**	**Target Recognition/Identification**	
Peptide libraries	Multiple (Proteases, inhibitors, B-cell and T-cell epitopes)	[155,156]
Carbohydrate libraries	Few	[157,158]
Aptamers	Multiple	[159]
Dendrimers	Few	[160,161]
Molecular imprints	Few	[162,163]
**Diagnostics**	**Target Quantification**	
Aptamers	Multiple (research/development stage)	[164,165]
**Therapeutics**	**Target Neutralization**	
Aptamers	Pegaptanib/ocular neo-vascularization	[166]

## Abbreviations

ELISA	Enzyme-linked immunosorbent assay
Fmoc	9-fluorenylmethyloxycarbonyl
ICC	Immunocytochemistry
IHC	Immunohistochemistry
IP	Immunoprecipitation
FC	Flow cytometry
HFMV	Hand foot mouth virus
scFv	single chain fragment variable
SPPS	Solid-phase peptide synthesis
PTM	Posttranslational modification
PAb	Polyclonal antibody
Mab	Monoclonal antibody
